# Differential signaling mechanisms regulate expression of CC chemokine receptor-2 during monocyte maturation

**DOI:** 10.1186/1476-9255-2-14

**Published:** 2005-10-31

**Authors:** Roderick J Phillips, Marin Lutz, Brett Premack

**Affiliations:** 1 Department of Physiology David Geffen School of Medicine at University of California, Los Angeles, Los Angeles, California, 90095 USA; 2 Jonsson Comprehensive Cancer Center, David Geffen School of Medicine at University of California, Los Angeles, Los Angeles, California, 90095 USA; 3 Department of Discovery Research, Intermune, 3280 Bayshore Blvd, Brisbane, California, 94005 USA; 4 Department of Technology Development, ChemoCentryx Inc., 1539 Industrial Road, San Carlos, California USA

**Keywords:** Human, Cellular Differentiation, Cell Surface Molecules, Gene Regulation

## Abstract

**Background:**

Peripheral blood monocytes and monocyte-derived macrophages are key regulatory components in many chronic inflammatory pathologies of the vasculature including the formation of atherosclerotic lesions. However, the molecular and biochemical events underlying monocyte maturation are not fully understood.

**Methods:**

We have used freshly isolated human monocytes and the model human monocyte cell line, THP-1, to investigate changes in the expression of a panel of monocyte and macrophage markers during monocyte differentiation. We have examined these changes by RT-PCR and FACS analysis. Furthermore, we cloned the CCR2 promoter and analyzed specific changes in transcriptional activation of CCR2 during monocyte maturation.

**Results:**

The CC chemokine receptor 2 (CCR2) is rapidly downregulated as monocytes move down the macrophage differentiation pathway while other related chemokine receptors are not. Using a variety of biochemical and transcriptional analyses in the human THP-1 monocyte model system, we show that both monocytes and THP-1 cells express high levels of CCR2, whereas THP-1 derived macrophages fail to express detectable CCR2 mRNA or protein. We further demonstrate that multiple signaling pathways activated by IFN-γ and M-CSF, or by protein kinase C and cytoplasmic calcium can mediate the downregulation of CCR2 but not CCR1.

**Conclusion:**

During monocyte-to-macrophage differentiation CCR2, but not CCR1, is downregulated and this regulation occurs at the level of transcription through upstream 5' regulatory elements.

## Background

Chemokines are a superfamily of small (8–10 kDa) proteins, which coordinate cellular responses to inflammation, insult or injury [1-4]. They also play a pivotal role in the regulation of leukocyte trafficking and extravasation through the luminal surface of endothelial cells into sites of tissue inflammation. The chemokine superfamily includes at least 20 receptors and more than 50 ligands [1-5]. The chemokine ligands can be separated into two major categories depending on whether they express a CC or CXC amino acid motif in their N-termini. This dichotomy appears to be functionally important since many CC chemokines preferentially target monocytes and T cells, while CXC chemokines such as IL-8 (CXCL8) tend to attract neutrophils. The CC chemokines bind to a family of G-protein coupled serpentine (seven transmembrane spanning) receptors, which are termed CC chemokine receptors (CCRs; [1,3,6]). Currently ten of the CC receptors have been identified and monocytes predominantly express three of them: CCR1, CCR2 and CCR5 [2,7,8]. These receptors can bind and signal to different CC chemokines including MCP-1 (CCL2), MIP-1α (CCL3) and RANTES (CCL3) [3,4,9] and these same chemokines are secreted by endothelial cells when activated by LDL or inflammatory cytokines [10-13] or when the endothelium is damaged [14,15].

Indeed, the recruitment of peripheral blood monocytes to the site of injured endothelium by pro-inflammatory chemokines is a key regulatory component in the formation of an atherosclerotic lesion [16,17]. The monocytes subsequently adhere to the endothelium and eventually migrate into the sub-intima [18,19]. Here, they receive a series of differentiation signals including macrophage-colony stimulating factor (M-CSF) and minimally oxidized LDL that enables them to mature into macrophages. These macrophages then engulf large quantities of cholesterol to become lipid-laden foam cells. And it is the accumulation of these foam cells that eventually leads to the formation of characteristic fatty streaks, intermediate lesions and fibrous plaques [20,21].

To date, though, the actual role of chemokines and their receptors in atherosclerosis has not been clearly established. However, recent studies using transgenic mouse models of atherosclerosis have provided convincing evidence that CCR2 is required for disease progression in apolipoprotein E-null mice [22,23]. In these animals, disruption of the CCR2 gene greatly decreases lesion formation without affecting plasma lipid or lipoprotein concentrations. Using a slightly different approach Rollins and colleagues have demonstrated that CCL2, the ligand for CCR2, plays an equally important role in the development of atherosclerosis in low-density lipoprotein receptor deficient mice [24,25]. Here, deletion of CCL2 leads to a significant reduction in lipid deposition within the aorta.

Despite the promising experimental results from these systems, relatively little is known about how the expression of chemokine receptor genes is regulated in normal or diseased human tissues. A recent paper by Yamamoto and colleagues [26] examined the basal regulatory mechanisms underlying expression of the CCR2 gene in the human monocyte cell line, THP-1. Indeed, this group characterized two key elements that seemed to be necessary and sufficient for the basal regulation of CCR2 expression: an Oct-1 binding sequence located 36 bp upstream of the TATA box and a tandem CAAT/enhancer-binding protein (C/EBP) binding sequence located, unusually, in the 5' UTR (at +50 to +77 bp). However, studies have not directly examined the molecular mechanisms by which basal expression of CCR2 is rapidly downregulated during the differentiation of monocytes into macrophages.

In an effort to address this issue, we have further developed a model of monocyte differentiation using THP-1 cells, which can be induced to mature into macrophages using either phorbol esters and ionomycin or a physiological combination of interferon-γ (IFN-γ) and M-CSF. In common with other studies, we report here that THP-1 cell maturation mediated by either high concentrations of PMA (50 nM) alone, or very low concentrations of PMA (1 nM) plus ionomycin (1 μM) is characterized by an increase in size, the development of an adherent phenotype and the up-regulation of a panel of differentiation markers [27-30]; in addition, CCR2, but not CCR1, was specifically down-regulated during differentiation. Modulation of CCR2 by PMA (50 nM), but not PMA (1 nM) plus ionomycin (1 μM), was found to be sensitive to inhibition by the broad-spectrum protein kinase inhibitor staurosporine. Furthermore, transient transfection of THP-1 cells with a CCR2-specific reporter construct indicated that PMA (50 nM) and PMA (1 nM) plus ionomycin (1 μM) mediated the downregulation of CCR2 through inhibition of CCR2-specific gene transcription. Moreover, physiological treatment of THP-1 monocytes with two known differentiation factors, IFN-γ and M-CSF, also promoted a differentiation phenotype essentially identical to that observed using pharmacologic stimuli. These data indicate that the activation of several intracellular signaling pathways selectively regulate the expression of CCR2 during monocyte maturation into macrophages.

## Materials and methods

### Cell lines

The THP-1 human monocytic cell line (ATCC) was grown in RPMI 1640 medium (GibcoBRL) containing 10 % fetal calf serum (FCS; GibcoBRL), 100 U/ml penicillin and 100 μg/ml streptomycin (GibcoBRL). The cells were maintained in culture at 37°C and 5% C0_2_. Typically, cells (7 × 10^6 ^per point) were stimulated with 50 nM phorbol myristate acetate (PMA; Sigma) or 1 nM PMA plus 1 μM ionomycin (Calbiochem) in the presence or absence of the PKC inhibitor staurosporine (Calbiochem).

### Isolation and culture of human peripheral blood monocytes

Peripheral blood mononuclear cells (PBMCs) were isolated from freshly prepared leukopacks (buffy coats) that were between 2–4 hours old. Briefly, 20 ml of blood from leukopacks were diluted using PBS (1:1) and layered over 15 ml of Ficoll-Paque PLUS (Amersham Pharmacia Biotech). Cells were then centrifuged at 400 × g for 20 minutes at room temperature. After this time, PBMCs were collected from the interphase and washed (× 2) with PBS and centrifuged at 150 × g for 10 minutes. Monocytes were further isolated from PBMCs using Percoll (Amersham Pharmacia Biotech) gradient centrifugation as previously described [31]. Lipid staining of the monocytes revealed that their purity was greater than 90%. Finally, the cells were resuspended and cultured at 10^6^/ml in RPMI 1640 supplemented with 10% autologous serum, penicillin and streptomycin (GibcoBRL).

### Cloning the CCR2 promoter

A 1335 bp fragment of the promoter from the *hCCR2 *gene was cloned into the pGL3 vector (Promega) using sequences determined by Yamamoto and colleagues [26]. This construct, termed pGL3-1335, contained the tandem C/EBP sites plus 1220 bp of the promoter sequence 5' of the transcriptional start site. The 5' primer contained a restriction site for *kpnI*, while the 3' primer contained a *HindIII *site. Each primer started with a 2 bp GC-rich clamp. The full primer sequences used are as follows:

pGL3-1335 5' CGGGTACCGCTGCTTTAGGTCCATTTACCCTC

pGL3-1335 3' GCAAGCTTATTGTACATTGGGTTGAGGTCTCC.

The genomic PCR was performed using an annealing temperature of 55°C (30 seconds) and an extension temperature of 72°C (2 minutes); 30 cycles of PCR were performed.

### RNA isolation and RT-PCR

Total RNA was isolated using TRIzol (Life Technologies) and by following the manufacturer's instructions. Briefly, cells were lyzed in TRIzol and then mixed with chloroform. The lysate was then centrifuged to separate RNA, DNA and protein. Total RNA, which is contained in the upper aqueous phase was recovered and mixed with isopropanol to precipitate the RNA. The RNA was finally washed in 75% ethanol to remove impurities and dissolved in water.

5 μg of RNA prepared in this way was then taken and DNase treated to remove further enzymatic contamination, before being reverse transcribed to cDNA using a ProSTAR First Strand RT-PCR kit from Stratagene and by following the manufacturer's instructions.

Subsequently, RT-PCR was performed under standard conditions using primers specific for CCR1, CCR2 and GAPDH. The primer sequences used here were:

CCR1 sense 5'GAAACTCCAAACACCACAGAGGAC

CCR1 antisense 5'TTCGTGAGGAAAGTGAAGGCTG

CCR2 sense 5'CCACATCTCGTTCTCGGTTTATCAG

CCR2 antisense 5'CGTGGAAAATAAGGGCCACAG

CCR3 sense 5'CACTAGATACAGTTGAGACCTTTGG

CCR3 antisense 5'GGTAAAGAGCACTGCAAAGAGTC

CCR4 sense 5'ACCCCACGGATATAGCAGATACC

CCR4 antisense 5'CGTCGTGGAGTTGAGAGAGTACTTG

CCR5 sense 5'GGAGCCCTGCCAAAAAATC

CCR5 antisense 5'CTGTATGGAAAATGAGAGCTGC

CCR6 sense 5'TGGCAAGGGGTATAATTTGGG

CCR6 antisense 5'GACAGTCTGGTACTTGGGTTCACAG

CCR7 sense 5'AGACAGGGGTAGTGCGAGGC

CCR7 antisense 5'GGATGGAGAGCACTGTGGCTAG

CCR8 sense 5'ACCTCAGTGTGACAACAGTGACCG

CCR8 antisense 5'ACCATCTTCAGAGGCCACTTGG

CCR9 sense 5'CACTGAGGATGCCGATTCTGAG

CCR9 antisense 5'CGAAATCTGCGTGGCAGTTC

CXCR1 sense 5'CAGATCCACAGATGTGGGA

CXCR1 antisense 5'GTTTGGATGGTAAGCCTGG

CXCR2 sense 5'AACATGGAGAGTGACAGC

CXCR2 antisense 5'GATGAGTAGACGGTCCTTC

CXCR3 sense 5'TCCTTGAGGTGAGTGACCA

CXCR3 antisense 5'GTATTGGCAGTGGGTGGCG

CXCR4 sense 5'AGTATATACACTTCAGATAAC

CXCR4 antisense 5'CCACCTTTTCAGCCAACAG

CXCR5 sense 5'CTGGACAGATTGGACAACTA

CXCR5 antisense 5'CATCACAACAACTCCCTGA

GAPDH sense 5'TCCATGACAACTTTGGTATCG

GAPDH antisense 5'GTCGCTGTTGAAGTCAGAGGA

The annealing temperature used for RT-PCR was 55°C for 30 seconds and the extension temperature was 72°C for 1 minute; typically 30 cycles of PCR were performed. Under these conditions the product sizes for CCR1, CCR2 and GAPDH were 567 bp, 580 bp and 420 bp respectively.

### Antibody staining and FACS analysis

THP-1 cells or PBMCs were resuspended in ice-cold staining buffer (PBS + 2% FCS + 0.1% sodium azide) and incubated with Fc block (Miltenyi Biotec) for 5 minutes at 4°C. Subsequently, primary antibodies were added (anti-CCR1, CCR2, CCR5, CCR7, CXCR2 and CXCR4; R&D Systems) at a final concentration of 0.5 μg/μl. The cells were then incubated at 4°C for 25 minutes, after which time they were washed twice in staining buffer. The secondary antibody used for these experiments was Alexa 488 (Molecular Probes) at a final concentration of 1 μg/μl. This time the cells were incubated at 4°C for 25 minutes in the dark. Following incubation with the secondary antibody, the cells were again washed twice, and then resuspended in 500 μl of staining buffer. Samples were finally analyzed on a FACScan flow cytometer (Becton Dickinson) using Cellquest 3.2.1f1 software. Peripheral blood monocytes, monocyte-derived macrophages and THP-1 cells were also stained for CD36, CD11b and CD68 (all purchased from BD Biosciences).

### Transient transfection using DEAE/Dextran

THP-1 cells, grown to a density of 5–8 × 10^5^/ml, were resuspended in Tris-buffered saline (TBS; 25 mM Tris.Cl, pH7.4, 137 mM NaCl, 5 mM KCl, 0.6 mM Na_2 _HPO4, 0.7 mM CaCl_2 _and 0.5 mM MgCl_2_). THP-1 cells (7 × 10^6 ^per point) were then added to 1 ml of TBS containing 5 μg of the CCR2 promoter-luciferase construct, 2 μg of the renilla control construct (pRL-SV40; Promega) and 500 μg/ml DEAE/Dextran (final concentration). This mixture was then left at room temperature for one hour. Next, DMSO was added to the cells drop-wise to a final concentration of 10% and incubated for 2 minutes at room temperature. Subsequently, the cells were washed twice in TBS, once in RPMI 1640 medium lacking FCS and antibiotics and once in RPMI 1640 complete medium. The cells were then resuspended in RPMI 1640 complete medium, stimulated with PMA and ionomycin (at the concentrations indicated) and finally incubated at 37°C and 5% CO_2 _for 48 hours.

After the 48-hour incubation period, cell extracts were made using the luciferase reporter lysis buffer (Promega). Each lysate was subsequently assayed in the dual luciferase reporter assay (Promega) following the manufacturer's instructions. Luciferase activity was determined using a Monolight series 2010 luminometer (Analytical Luminescence Laboratory) and then normalized to the renilla control.

## Results

### Freshly isolated monocytes selectively downregulate CCR2, but not CCR1, in culture

**Figure 1:**
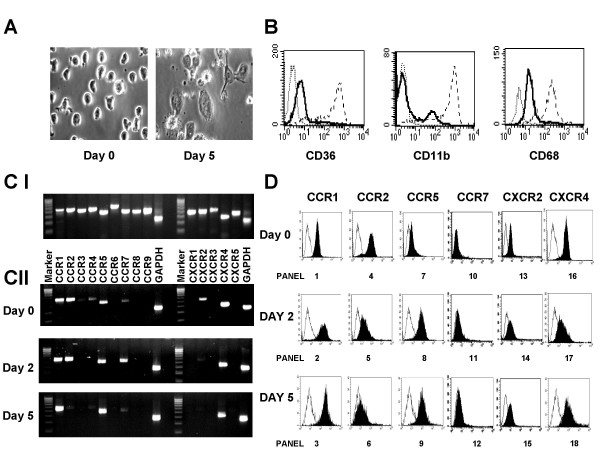
**Macrophage-derived monocytes selectively downregulate CCR2, but not CCR1, during differentiation**. (a). Changes in morphology between freshly isolated monocytes (left panel) and cells cultured for 5 days (right panel) were determined using a Nikon Diaphot Camera set up and Axon Imaging Workbench software. Magnification is at 60 ×. (b). Freshly isolated monocytes were either cultured for 5 days (broken line) or immediately stained (solid line) for a panel of macrophage markers: CD36 (left panel), CD11b (middle panel) or CD68 (right panel). Dotted histograms represent the isotype controls. (c). Panel I. Genomic DNA was prepared from freshly isolated monocytes and assayed for germ line expression of chemokine receptors CCR1-CCR9 and CXCR1-CXCR5 by PCR using primers designed in-house. Note each primer pair amplified a single product only, thus confirming that the primers are functional and specific. Panel II. Messenger RNA was prepared from freshly isolated monocytes (upper panel) and cells that had been cultured for either 2 days (middle panel) or 5 days (lower panel). Subsequently, RT-PCR was performed using primers for chemokine receptors CCR1-CCR9, CXCR1-CXCR5 and GAPDH. Marker is a 100 bp DNA ladder. Similar results were obtained in three other experiments. (d). Freshly isolated monocytes (upper panel plots 1, 4, 7, 10, 13 and 16) and cells that had been cultured for either 2 days (middle panel plots 2, 5, 8, 11, 14 and 17) or 5 days (lower panel plots 3, 6, 9, 12, 15 and 18) were stained for CCR1, CCR2, CCR5, CCR7, CXCR2 and CXCR4. Cells were then analyzed for changes in chemokine receptor expression by flow cytometry. Similar results were obtained in three other experiments.

Human monocytes were isolated from blood leukopacks and placed in culture for up to 5 days (Figure 1). During this time these cells underwent changes in both morphology and gene expression. Freshly isolated monocytes initially appeared small and round, but after 5 days in culture they became adherent, and increased in both size and granularity (Figure 1A). Next, we analyzed changes in the expression of the macrophage differentiation markers CD11b, CD36 and CD68 (Figure 1B). We found that monocytes cultured for 5 days upregulated expression of the integrin CD11b and the scavenger receptors CD36 and CD68, consistent with a change in phenotype from monocyte to macrophage (Figure 1B). Next, we wanted to examine changes in the expression of chemokine receptors as monocytes differentiated into macrophages. Using primers specific for CXCR1-5 and CCR1-CCR9, we performed semi-quantitative analysis of receptor mRNA expression (Figure 1C). Initially, however, we determined the efficacy and specificity of the primers by analyzing genomic DNA samples prepared from freshly isolated monocytes (Figure 1C, panel I). In all cases a single band of the anticipated size was observed indicating that the primers were specific for the desired chemokine receptor. This data further suggested that a lack of chemokine receptor expression observed in freshly isolated monocytes and monocytes cultured for up to five days was a true result, rather than as a reflection of inappropriate primer design. Subsequently, we performed semi-quantitative analysis of receptor mRNA expression on freshly isolated monocytes and monocytes cultured for up to five days (Figure 1C, panel II). Under these conditions, freshly isolated monocytes showed strong expression of CCR1, CCR2, CCR5, CXCR2 and CXCR4 mRNAs, and trace levels of CCR4 and CCR7 mRNA. Expression of CCR1, CCR2, CCR5, and CXCR4 mRNAs remained elevated after two days in culture, while that of CXCR2 decreased and that of CCR7 temporarily increased. However, after five days in culture CCR2 mRNA expression but not that of CCR1, CCR5 or CXCR4 was dramatically downregulated (Figure 1C, panel II). Indeed, levels of CCR5 and CCR1 mRNA actually increased over those observed in freshly isolated monocytes. To confirm the specificity of this effect we subsequently compared cell surface expression of these chemokine receptors in cultured monocytes and freshly isolated monocytes by flow cytometry (Figure 1D). In agreement with our mRNA data, expression of CCR2 protein, but not CCR1, CCR5 and CXCR4 was rapidly downregulated during monocyte maturation. Negligible cell surface expression of CCR7 protein was observed at any of the time points examined, while CXCR2 cell surface expression remained curiously elevated despite downregulation of CXCR2 mRNA, suggesting that the half-life of this protein is actually quite long (Figure 1D).

These results indicate that one consequence of monocyte maturation is the selective downregulation of CCR2 gene expression followed by a loss of CCR2 protein from the surface of the cell. While the actual physiological role of this process is unknown, it is likely that CCR2 down-regulation may be involved in restricting 'reverse-migration' of differentiated monocytes back into the blood stream, and thus facilitating capture within the tissues.

### PMA-treatment of monocytes induces selective downregulation of CCR2

**Figure 6:**
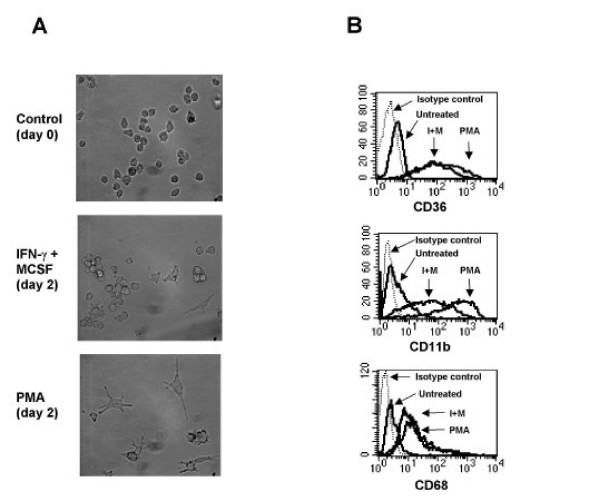
**IFN-γ plus M-CSF promotes a similar differentiation phenotype to that observed using pharmacologic stimuli**. (a). THP-1 cells were either left untreated (upper panel) or treated with 500 U/ml IFN-γ plus 5 ng/ml M-CSF (middle panel) or 50 nM PMA (lower panel) for 48 hours. Subsequently, the cells were photographed using a Nikon Diaphot Camera set up and Axon Imaging Workbench software. Magnification is at 40 ×. (b). THP-1 cells were either left untreated or treated for 48 hours with either 50 nM PMA (PMA) or 500 U/ml IFN-γ plus 5 ng/ml M-CSF (I+M) as indicated. Subsequently, these cells were stained with antibodies to macrophage markers CD36 (upper panel), CD11b (middle panel) and CD68 (lower panel) and then analyzed by flow cytometry.

Based on the above results we decided to further examine the regulation of CCR2 expression in monocyte maturation using the human monocyte cell line, THP-1 and CCR1 as a control. Treatment of these cells with the PKC-activating phorbol ester PMA for 48 hours is a widely accepted procedure for maturing monocytes [27,28]. Cells treated in this way undergo phenotypic changes consistent with their maturation into macrophages [27-30] (also compare Figures 1 and 6).

**Figure 2:**
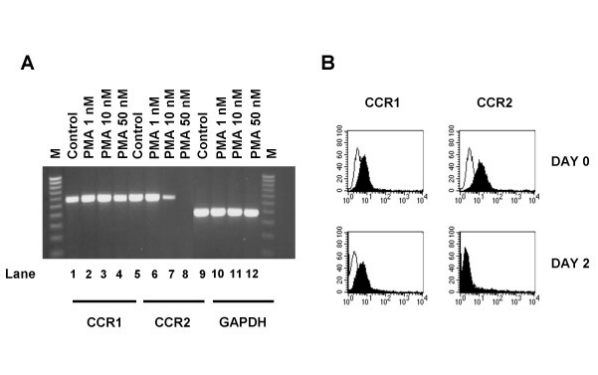
**PMA induces a dose-dependent selective downregulation of CCR2**. (a). THP-1 cells were either untreated (lanes 1, 5 and 9) or treated with PMA at 1 nM (lanes 2, 6 and 10), 10 nM (lanes 3, 7 and 11) or 50 nM (lanes 4, 8 and 12) for 48 hours. Messenger RNA was then prepared and RT-PCR performed using primers for CCR1 (lanes 1–4), CCR2 (lanes 5–8) and GAPDH (lanes 9–12). M is a 100 bp DNA ladder. Similar results were obtained in seven other experiments. (b). THP-1 cells were either left untreated or stimulated with PMA (50 nM) for the times indicated. Subsequently the cells were introduced into a FACScan flow cytometer to measure cell surface expression of CCR1 (left panel) or CCR2 (right panel).

Next, we wanted to determine how treatment of the monocyte cell line, THP-1, with PMA affected the expression of CCR2 in these cells. Thus, monocytes were stimulated with PMA (at the concentrations indicated) for 48 hours and RNA prepared as described above. Our results (Figure 2A) show that CCR2 was selectively down-regulated in a dose dependent manner, whereas expression of CCR1 (the other main CC receptor on monocytes) and the house-keeping gene GAPDH remained unaffected. PMA (50 nM) was sufficient to completely abrogate CCR2 expression (Figure 2A, lane 8), whilst 10 nM PMA reduced expression of this chemokine receptor by approximately 75% (Figure 2A, lane 7). Treatment of THP-1 cells with 1 nM PMA did not affect expression of CCR2 (Figure 2A, lane 6).

Subsequently, we examined whether PMA modulated the cell surface expression of CCR1 and CCR2 by FACS analysis. THP-1 cells were again stimulated with PMA (50 nM) for the times indicated, before being stained with the appropriate antibodies and then analyzed by flow cytometry (Figure 2B). Whereas the levels of CCR1 remained high throughout the duration of the experiment, CCR2 protein expression decreased dramatically. The majority of the expression was lost by 24 hours and by 48 hours virtually no CCR2 was found on the surface of the cultured THP-1 cells (compare Figure 2B, left and right panels). Thus, THP-1 cells treated with PMA (50 nM) mimics the differentiation process observed in cultured monocytes.

### Two distinct signal transduction pathways regulate CCR2 expression during monocyte maturation

**Figure 3:**
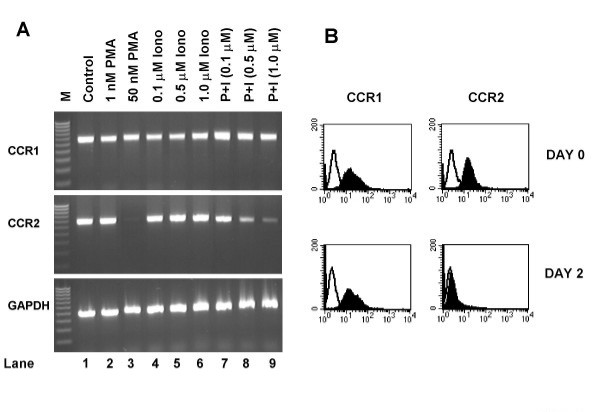
**Sub-optimal concentrations of PMA, together with a modest calcium signal, also modulate CCR2**. (a). THP-1 cells were either unstimulated (lane1) or treated with PMA 1 nM (lane 2) or 50 nM (lane 3) for 48 hours. Alternatively, the cells were treated with increasing concentrations of the calcium ionophore ionomycin alone (lanes 4–6) or in combination with PMA 1 nM (lanes 7–9) also for 48 hours. Messenger RNA was then prepared and RT-PCR performed using primers for CCR1 (upper panel), CCR2 (middle panel) and GAPDH (lower panel). M represents markers, which are a 100 bp ladder. Similar results were obtained in four other experiments. (b). THP-1 cells were either left untreated or stimulated with PMA (1 nM) and ionomycin (1 μM) for the times indicated. Subsequently the cells were stained for expression of CCR1 (left panel) or CCR2 (right panel) and analyzed by flow cytometry.

Our initial observations suggested that while PMA (50 nM) completely abrogated CCR2 expression, sub-optimal concentrations of this phorbol ester (1 nM) had no effect (Figure 2A). We wondered, therefore, whether the addition of a calcium signal (such as ionomycin) together with the sub-optimal concentration of PMA might provide a sufficiently strong stimulus to affect the expression of CCR2. Thus, we incubated monocytes with PMA (1 nM) and ionomycin at the concentrations indicated for 48 hours, and then analyzed CCR2 expression. Our data indicated that ionomycin alone does not affect expression of CCR2 (Figure 3A, middle panel, lanes 4–6). However, in the presence of a sub-optimal PMA signal (1 nM), there was a selective dose-dependent reduction in CCR2 expression (Figure 3A, middle panel, lanes 7–9). At the same time, similar concentrations of PMA and ionomycin did not affect the levels of CCR1 nor GAPDH (Figure 3A upper and lower panels). Monocytes treated with PMA (1 nM) plus ionomycin (1 μM) were also observed to adopt an adherent phenotype and to increase in size similar to the changes in morphology observed in freshly isolated monocytes (data not shown). Furthermore, cell surface expression of CCR2, but not CCR1, was found to be downregulated in the presence of PMA (1 nM) plus ionomycin (1 μM) after 48 hours (Figure 3B). Thus, sub-optimal concentrations of PMA together with a modest calcium signal combine to mediate a maturation phenotype in monocytes that also includes the selective downregulation of CCR2.

**Figure 4:**
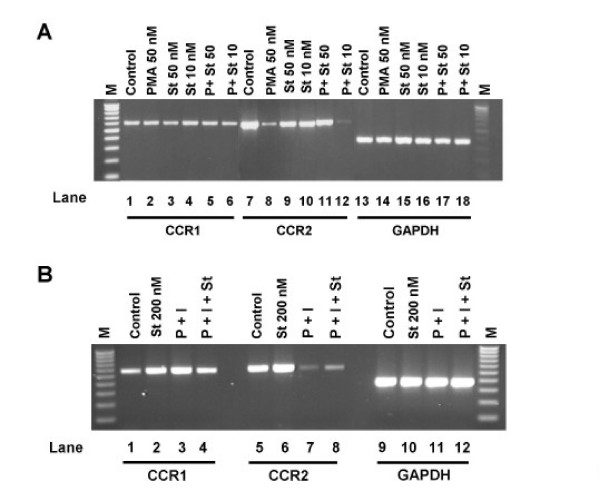
**The PKC-inhibitor staurosporine blocks PMA, but not PMA plus ionomycin, induced downregulation of CCR2**. (a). THP-1 cells were either untreated (lanes 1, 2, 7, 8, 13 and 14) or preincubated with 50 nM staurosporine (lanes 3, 5, 9, 11, 15 and 17) or 10 nM staurosporine (lanes 4, 6, 10, 12, 16 and 18) for 2 hours. Subsequently, the cells were stimulated with 50 nM PMA (lanes 2, 5, 6, 8, 11, 12, 14, 17 and 18) for a further 46 hours. Messenger RNA was then prepared and RT-PCR performed using primers for CCR1 (lanes 1–6), CCR2 (lanes 7–12) and GAPDH (lanes 13–18). M is a 100 bp DNA ladder. Similar results were obtained in three other experiments. (b). THP-1 cells were either untreated (lanes 1, 3, 5, 7, 9 and 11) or preincubated with 200 nM staurosporine (lanes 2 and 4, 6 and 8 and 10 and 12) for 2 hours. Subsequently the cells were stimulated with a combination of 1 nM PMA and 1 μM ionomycin (lanes 3 and 4, 7 and 8 and 11 and 12) for a further 46 hours. Messenger RNA was then prepared and RT-PCR performed using primers for CCR1 (lanes 1–4), CCR2 (lanes 5–8) and GAPDH (lanes 9–12). M is a 100 bp DNA ladder. Similar results were obtained in three other experiments.

To determine whether the selective downregulation of CCR2 observed in PMA versus PMA plus ionomycin treated cells represented the same or two different signaling pathways, we performed an experiment using the broad-spectrum kinase inhibitor, staurosporine (Figure 4). We preincubated THP-1 cells with staurosporine at the concentrations indicated for two hours, and then stimulated with either PMA (50 nM; Figure 4A) or PMA (1 nM) plus ionomycin (1 μM; Figure 4B) for 48 hours. Staurosporine alone (at concentrations up to 200 nM) did not significantly inhibit expression of CCR2 (Figure 4A, lanes 9 and 10 and Figure 4B, lane 6) nor CCR1 (Figure 4A, lanes 3 and 4 and Figure 4B, lane 2). Furthermore, the inhibitor did not abrogate the downregulation of CCR2 mediated by PMA plus ionomycin (Figure 4B, compare lanes 7 and 8). In contrast, staurosporine at 50 nM, but not at 10 nM, blocked the loss of CCR2 in PMA (50 nM) treated cells (Figure 4A, compare lanes 7, 8, 11 and 12).

Thus, these results identify at least two possible signal transduction pathways present in monocytes that could regulate the expression of CCR2 during monocyte differentiation.

### CCR2 expression is regulated at the level of transcription

Having established that CCR2 is down-regulated during monocyte differentiation, we next wanted to determine whether the regulation occurs at the level of RNA stability or at the level of transcription. We, therefore, cloned a 1335 bp fragment of the CCR2 promoter using the sequence described by Yamamoto and colleagues [26]. This fragment was then subcloned into the mammalian expression vector pGL3 upstream of the luciferase gene, generating the pGL3-1335 construct. In addition to the sequences upstream of the TATA box, pGL3-1335 included 115 bp of the 5'UTR, which contains the two tandem C/EBP repeats that are thought to be necessary for the basal expression of the CCR2 gene [26].

**Figure 5:**
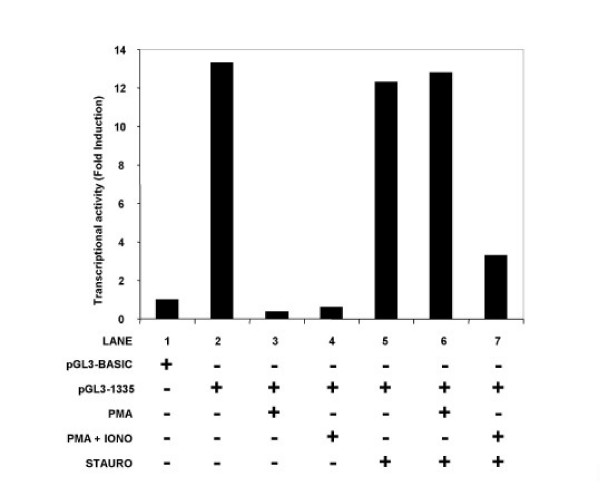
**Staurosporine blocks PMA, but not PMA plus ionomycin, induced downregulation of CCR2 promoter activity**. THP-1 cells were transfected with either 5 μg of vector alone (pGL3-basic; lane 1) or with 5 μg of the pGL3-1335 construct (lanes 2–7). In addition, each sample was also co-transfected with 2 μg of pRL-SV40 (renilla) to act as an internal control. Cells were then either left untreated (lanes 1–4) or pretreated with staurosporine (100 nM) for 2 hours (lanes 5–7). Next, the THP-1 cells were stimulated with a combination of PMA alone (lanes 3 and 6) or PMA plus ionomycin (lanes 4 and 7) for a further 46 hours. Subsequently, cell extracts were prepared and assayed for both luciferase and renilla activity. After normalization to the renilla control, the CCR2 transcriptional activity was determined relative to the pGL3-basic vector, which was arbitrarily assigned a value of 1. Similar results were obtained in two other experiments

Subsequently, we transfected this construct into the THP-1 cells using DEAE/dextran and either left the cells untreated, or treated them with PMA (50 nM), or PMA (1 nM) plus ionomycin (1 μM) for 48 hours in the presence or absence of staurosporine (100 nM). Cells were then lyzed and assayed for transcriptional activity. Our results showed that the pGL3-1335 construct, itself, gave a 13-fold induction over the background construct lacking the CCR2 promoter (Figure 5, compare lanes 1 and 2). Furthermore, both PMA and PMA plus ionomycin strongly abrogated this transcriptional activity (Figure 5 lanes 3 and 4) suggesting that the dual signal transduction pathways activated by PMA and PMA plus ionomycin mediated regulation of CCR2 expression at the level of transcription. In the presence of staurosporine, inhibition of CCR2 promoter activity mediated by PMA, but not PMA (1 nM) plus ionomycin (1 μM), was abrogated (Figure 5, compare lanes 6 and 7). Thus, these data indicate that the PMA (but not the PMA plus ionomycin) mediated inhibition of CCR2 promoter activity is ultimately regulated by one or more staurosporine-sensitive transcription factors.

### Treatment with IFN-γ and M-CSF produces a similar differentiation phenotype to that seen with PMA and ionomycin

The above results reflect a phenotype induced by pharmacologic agents and we next wanted to ensure that this phenotype is applicable to physiologic agents also. To that end, THP-1 cells treated with IFN-γ plus M-CSF have already been shown to promote monocyte maturation, although it has yet to be confirmed that these agents regulate CCR2 expression at the level of transcription [32]. Initially, though, we wanted to demonstrate that monocytes treated with IFN-γ plus M-CSF showed changes in morphology similar to that observed with freshly isolated monocytes (compare Figures 1 and 6). After 48 hours treatment with IFN-γ plus M-CSF, monocytes became adherent and increased in size similar to that observed for freshly isolated monocytes in culture (compare Figure 1A and Figure 6A middle panel). PMA-treated monocytes also underwent similar changes in morphology (Figure 6A, lower panel). Furthermore, flow cytometric studies revealed that monocytes treated with either IFN-γ plus M-CSF or PMA strongly upregulated the macrophage maturation markers CD11b, CD36 and CD68 (Figure 6B). Similar results were observed for cells treated with PMA plus ionomycin (data not shown). Thus, monocytes treated with a panel of physiologic and pharmacologic stimuli promote maturation to the macrophage phenotype as determined by changes in morphology and upregulation of macrophage maturation markers.

**Figure 7:**
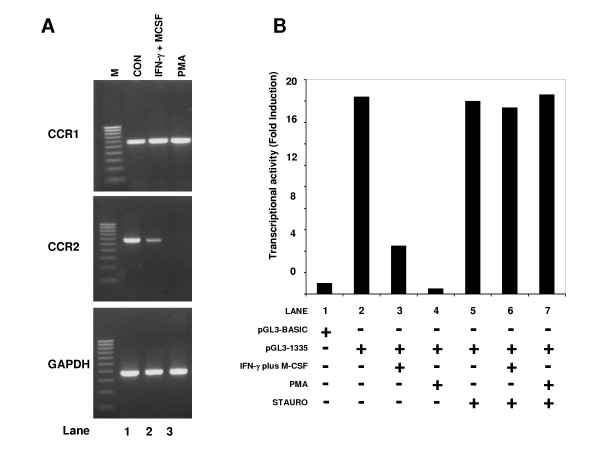
**IFN-γ plus M-CSF promotes specific down-regulation of CCR2**. (a). THP-1 cells were either untreated (lane 1, upper, middle and lower panels) or treated with 500 U/ml IFN-γ plus 5 ng/ml M-CSF (lane 2 upper, middle and lower panels) or 50 nM PMA (lane 3 upper, middle and lower panels) for 48 hours. Messenger RNA was then prepared and RT-PCR performed using primers for CCR1 (upper panel), CCR2 (middle panel) and GAPDH (lower panel). M is a 100 bp DNA ladder. Similar results were obtained in three other experiments. (b). THP-1 cells were transfected with either 5 μg of vector alone (pGL3-basic) or with 5 μg of the pGL3-1335 construct. In addition, each sample was also transfected with 2 μg of pRL-SV40 (renilla) to act as an internal control. Cells were then either left untreated or treated with either 500 U/ml IFN-γ plus 5 ng/ml M-CSF or 50 nM PMA. Subsequently, cell extracts were prepared and assayed for both luciferase and renilla activity. After normalization to the renilla control, CCR2 transcriptional activity was calculated relative to the pGL3-basic vector, which was arbitrarily assigned a value of 1. Similar results were obtained in two other experiments.

Next, we wanted to determine whether IFN-γ plus M-CSF induced the differentiation-associated downregulation of CCR2 (Figure 7). Therefore, monocytes were treated with IFN-γ (500 U/ml) plus M-CSF (5 ng/ml) for 48 hours and CCR2 mRNA was examined (Figure 7A). Our results showed that IFN-γ plus M-CSF did selectively downregulate CCR2, but not CCR1 in a manner analogous to that observed for PMA and PMA plus ionomycin (Figure 7A upper and middle panels). A similar pattern was also observed when transcriptional activity was examined (Figure 7B). Here, PMA completely down-modulated CCR2 transcription, while the combined effects of IFN-γ plus M-CSF reduced this activity by approximately 70%. In the presence of staurosporine, the inhibition of CCR2 promoter activity mediated by IFN-γ (500 U/ml) plus M-CSF (5 ng/ml) was abrogated in a manner analogous to that observed for PMA (Figure 7B lanes 6 and 7).

Taken together, these data suggest that PMA (50 nM), PMA plus ionomycin and IFN-γ plus M-CSF mediate similar changes in the monocyte phenotype during maturation of these cells. Thus, the monocyte cell line, THP-1, is a useful model system with which to investigate the underlying regulatory mechanisms governing chemokine receptor expression during monocyte differentiation.

## Discussion

In this paper we demonstrate that a major consequence of monocyte maturation into macrophages is the selective downregulation of the chemokine receptor, CCR2, but not the related CCR1. We have further shown that there are multiple stimuli, which can selectively down-modulate CCR2 expression, including high concentrations of PMA (50 nM), or low PMA (1 nM) plus ionomycin (1 μM), or IFN-γ (500 U/ml) plus M-CSF (5 ng/ml). Each of these stimuli regulate the expression of CCR2 at the level of transcription, although it appears that at least two different signal transduction pathways are involved based on the ability of staurosporine to interfere with these processes. Treatment of THP-1 monocytes with staurosporine abrogated the ability of PMA and IFN-γ plus M-CSF to downregulate CCR2. By contrast, staurosporine was unable to block PMA plus ionomycin mediated downregulation of CCR2 expression. Thus, this study provides evidence that there is dynamic and selective regulation of the CCR2 gene during monocyte differentiation.

Our results indicate that treatment of THP-1 cells with either PMA alone (50 nM) or PMA (1 nM) plus ionomycin (1 μM) promotes a differentiation phenotype that is characterized by morphological changes and altered CCR2 gene expression. Indeed, these observations have already been noted by other researchers studying monocyte differentiation [27,28,32]. In particular, we show that THP-1 cells rapidly become adherent and their morphology changes from the typical round shape of monocytes to spindle-shaped cells with pseudopodia, which are characteristic of macrophages. At the same time there was also an increase in the size and granularity of the cells. In addition, we demonstrated an up-regulation in expression of genes associated with monocyte differentiation, notably CD11b, CD36 and CD68. Concomitantly, the expression of CCR2, but not CCR1, was selectively downregulated, suggesting that the loss of this chemokine receptor is a consequence of monocyte differentiation. This downregulation was observed at the level of cell surface receptor expression, mRNA expression, and transcription. Clearly, these are specific regulatory events since the levels of CCR1 mRNA are not affected by either combination of pharmacologic agents.

However, when THP-1 cells were treated with PMA (50 nM) or PMA plus ionomycin in the presence of staurosporine, differential results were obtained: PMA-mediated modulation of CCR2 was sensitive to the inhibitory effects of staurosporine (50 nM), whereas staurosporine concentrations as high as 200 nM failed to block PMA plus ionomycin-induced downregulation of CCR2. Staurosporine alone did not promote the loss of either CCR2 or CCR1. These results indicate that staurosporine defines a dichotomy in the regulation of CCR2 expression by PMA (50 nM) versus PMA plus ionomycin that had not previously been appreciated.

Staurosporine, itself, is a broad-spectrum inhibitor of protein kinases including PKA, PKC, and PKG. PMA has classically been shown to act almost exclusively through PKC and this would explain why staurosporine was able to block the PMA-induced downregulation of CCR2. By inference, PMA plus ionomycin would appear to act through a signal transduction pathway that is not inhibited by staurosporine and presumably this means that second messengers other than PKA, PKC and PKG are involved. To that end, calcineurin, a calcium-sensitive phosphatase may be a target for PMA plus ionomycin [33]. An increase in the intracellular calcium concentration (such as that afforded by the presence of ionomycin) promotes a conformational change in calcineurin, which then dephosphorylates and activates the transcription factor NFAT facilitating its translocation to the nucleus. In addition, it has been shown that PMA enhances the calcium sensitivity of NFAT, thus creating a synergistic signal [33,34]. This synergy may result from de novo synthesis and post-translational modification of another transcription factor termed activating protein-1, AP-1 [33,34]. Indeed, NFAT proteins show a characteristic ability to co-operate with AP-1 in DNA-binding and transactivation [33,34]. Interestingly, in the region of the CCR2 promoter that we cloned there are two putative binding sites for AP-1 (core binding motif TGA(C/G)TCA) and three putative binding sites for NFAT (core binding motif GGAAA) as determined by the MatInspecter transcription factor binding site analysis program. It has also been suggested that additional transcription factors including OCT1 and C/EBP can act synergistically with NFAT and again there are multiple binding sites for each of these DNA-binding proteins in the CCR2 promoter, although at this stage we have no evidence to suggest that they are involved in the physiological regulation of CCR2 gene expression.

A requirement for co-operation and cross-talk between these two pharmacologic agents is further supported by the fact that ionomycin alone (at concentrations as high as 1 μM) was unable to down-modulate CCR2.

Some reports have suggested that CCL2 could be involved in the early stages of CCR2 protein down-modulation, while other studies indicate that the differentiation process itself, is a major factor in the selective loss of CCR2 gene expression [8,32]. Numerous cytokines are known to be involved in monocyte activation and differentiation, among them M-CSF and IFN-γ [32,35,36]. M-CSF is a lineage-specific hematopoetic growth factor that stimulates monocyte differentiation [35,36]. The *c-fms *proto-oncogene encodes a high affinity receptor for M-CSF [37] and it has been shown that THP-1 cells express this protein and that it is up-regulated during differentiation. However, cells stimulated with M-CSF alone for 48 hours did not lose expression of CCR2 (data not shown).

Conversely, IFN-γ alone, which is constitutively expressed by monocyte lineage cells and which promotes maturation of monocytes to macrophages [38], did significantly reduce expression of CCR2, although the cells did not become adherent and neither did they change their morphology (data not shown). Interestingly, IFN-γ has been demonstrated to up-regulate levels of M-CSF in monocytes during maturation [38] and when both IFN-γ and M-CSF were added, THP-1 cells did become adherent, changed their morphology and selectively lost CCR2, but not CCR1 – all of which are characteristics of the monocyte differentiation phenotype. These results are in keeping with the studies published by Tangirala and colleagues, who reported similar phenomena in THP-1 cells [32]. In addition, our studies also demonstrated that the regulatory effects mediated by IFN-γ plus M-CSF occurred at the level of transcription, where a significant down-regulation in CCR2 promoter activity was observed. Moreover, in the presence of staurosporine, IFN-γ plus M-CSF was unable to down-regulate levels of CCR2. This result probably reflects the fact that IFN-γ signals extensively through the JAK-STAT pathway, and studies have suggested that staurosporine can block phosphorylation of Janus kinases [39,40]. In addition, we have found two putative binding sites in the CCR2 promoter for STAT transcription factors which would further support the contention that these transcription factors may be important in the regulation of IFN-γ mediated downregulation of CCR2.

## Conclusion

This study demonstrates that expression of the chemokine receptor CCR2 is exquisitely correlated with monocyte maturation. Freshly isolated monocytes express high levels of both CCR2 RNA and protein, whereas monocyte-derived macrophages express neither CCR2 RNA nor protein. Conversely, levels of the closely-related chemokine receptor CCR1 remained stable and elevated throughout monocyte maturation. An analysis of the biochemical and molecular mechanisms underlying the regulated expression of CCR2 revealed the existence of several signaling pathways that selectively down-modulate CCR2 gene expression during monocyte differentiation; this expression was largely regulated at the level of transcription. Signaling through PMA and IFN-γ plus M-CSF, but not PMA plus ionomycin was abrogated by prior treatment of the THP-1 cells with staurosporine. Although the physiological role of this process is not well understood, it is likely that CCR2 down-regulation may be involved in restricting the 'reverse-migration' of differentiated monocytes back into the blood stream. This in turn facilitates the retention of differentiated monocytes within inflamed tissues. Thus, by improving our understanding of the regulatory mechanisms that govern CCR2 expression on monocyte lineage cells, we can better appreciate how monocyte recruitment and activation is controlled during chronic inflammatory pathologies such as atherosclerosis.

## Competing interests

Brett Premack is currently employed as the Director of Technology at Chemocentryx. Dr. Premack is the holder of stocks within this company. This company investigates the role of chemokines and their receptors as potential therapeutics. One of these projects is to investigate the role of CCR2 antagonists in cardiovascular disease and a phase I clinical trial is ongoing. At the time this study was undertaken Dr. Premack was an unpaid consultant for Chemocentryx. Neither Roderick Phillips nor Marin Lutz has a competing interest in this work.

## Authors' contributions

RJP wrote the manuscript and performed all of the experiments except Figure 1A and 1C. ML performed experiments featured in Figure 1A and 1C. BP conceived of the study, and participated in its design and coordination and helped to draft the manuscript. All authors read and approved the final manuscript.

RJP was supported by the American Heart Association grant 9960044Y

## References

[B1] Rossi D, Zlotnik A (2000). The biology of chemokines and their receptors. Annu Rev Immunol.

[B2] Murphy PM, Baggiolini M, Charo IF, Hebert CA, Horuk R, Matsushima K, Miller LH, Oppenheim JJ, Power CA (2000). International union of pharmacology. XXII. Nomenclature for chemokine receptors. Pharmacol Rev.

[B3] Premack BA, Schall TJ (1996). Chemokine receptors: gateways to inflammation and infection. Nat Med.

[B4] Baggiolini M (1998). Chemokines and leukocyte traffic. Nature.

[B5] Murphy PM (2002). International Union of Pharmacology. XXX. Update on chemokine receptor nomenclature. Pharmacol Rev.

[B6] Power CA, Wells TN (1996). Cloning and characterization of human chemokine receptors. Trends Pharmacol Sci.

[B7] Charo IF, Myers SJ, Herman A, Franci C, Connolly AJ, Coughlin SR (1994). Molecular cloning and functional expression of two monocyte chemoattractant protein 1 receptors reveals alternative splicing of the carboxyl-terminal tails. Proc Natl Acad Sci U S A.

[B8] Fantuzzi L, Borghi P, Ciolli V, Pavlakis G, Belardelli F, Gessani S (1999). Loss of CCR2 expression and functional response to monocyte chemotactic protein (MCP-1) during the differentiation of human monocytes: role of secreted MCP-1 in the regulation of the chemotactic response. Blood.

[B9] Neote K, DiGregorio D, Mak JY, Horuk R, Schall TJ (1993). Molecular cloning, functional expression, and signaling characteristics of a C-C chemokine receptor. Cell.

[B10] Brown Z, Gerritsen ME, Carley WW, Strieter RM, Kunkel SL, Westwick J (1994). Chemokine gene expression and secretion by cytokine-activated human microvascular endothelial cells. Differential regulation of monocyte chemoattractant protein-1 and interleukin-8 in response to interferon-gamma. Am J Pathol.

[B11] Goebeler M, Yoshimura T, Toksoy A, Ritter U, Brocker EB, Gillitzer R (1997). The chemokine repertoire of human dermal microvascular endothelial cells and its regulation by inflammatory cytokines. J Invest Dermatol.

[B12] Marfaing-Koka A, Devergne O, Gorgone G, Portier A, Schall TJ, Galanaud P, Emilie D (1995). Regulation of the production of the RANTES chemokine by endothelial cells. Synergistic induction by IFN-gamma plus TNF-alpha and inhibition by IL-4 and IL-13. J Immunol.

[B13] Martin T, Cardarelli PM, Parry GC, Felts KA, Cobb RR (1997). Cytokine induction of monocyte chemoattractant protein-1 gene expression in human endothelial cells depends on the cooperative action of NF-kappa B and AP-1. Eur J Immunol.

[B14] Kumar AG, Ballantyne CM, Michael LH, Kukielka GL, Youker KA, Lindsey ML, Hawkins HK, Birdsall HH, MacKay CR, LaRosa GJ, Rossen RD, Smith CW, Entman ML (1997). Induction of monocyte chemoattractant protein-1 in the small veins of the ischemic and reperfused canine myocardium. Circulation.

[B15] Wysocki SJ, Zheng MH, Smith A, Lamawansa MD, Iacopetta BJ, Robertson TA, Papadimitriou JM, House AK, Norman PE (1996). Monocyte chemoattractant protein-1 gene expression in injured pig artery coincides with early appearance of infiltrating monocyte/macrophages. J Cell Biochem.

[B16] Ross R (1993). The pathogenesis of atherosclerosis: a perspective for the 1990s. Nature.

[B17] Berliner JA, Navab M, Fogelman AM, Frank JS, Demer LL, Edwards PA, Watson AD, Lusis AJ (1995). Atherosclerosis: basic mechanisms. Oxidation, inflammation, and genetics. Circulation.

[B18] Cai JP, Hudson S, Ye MW, Chin YH (1996). The intracellular signaling pathways involved in MCP-1-stimulated T cell migration across microvascular endothelium. Cell Immunol.

[B19] Randolph GJ, Furie MB (1995). A soluble gradient of endogenous monocyte chemoattractant protein-1 promotes the transendothelial migration of monocytes in vitro. J Immunol.

[B20] Ross R (1995). Cell biology of atherosclerosis. Annu Rev Physiol.

[B21] Lusis AJ (2000). Atherosclerosis. Nature.

[B22] Boring L, Gosling J, Cleary M, Charo IF (1998). Decreased lesion formation in CCR2-/- mice reveals a role for chemokines in the initiation of atherosclerosis. Nature.

[B23] Charo IF, Peters W (2003). Chemokine receptor 2 (CCR2) in atherosclerosis, infectious diseases, and regulation of T-cell polarization. Microcirculation.

[B24] Gu L, Okada Y, Clinton SK, Gerard C, Sukhova GK, Libby P, Rollins BJ (1998). Absence of monocyte chemoattractant protein-1 reduces atherosclerosis in low density lipoprotein receptor-deficient mice. Mol Cell.

[B25] Rollins BJ (2001). Chemokines and atherosclerosis: what Adam Smith has to say about vascular disease. J Clin Invest.

[B26] Yamamoto K, Takeshima H, Hamada K, Nakao M, Kino T, Nishi T, Kochi M, Kuratsu J, Yoshimura T, Ushio Y (1999). Cloning and functional characterization of the 5'-flanking region of the human monocyte chemoattractant protein-1 receptor (CCR2) gene. Essential role of 5'-untranslated region in tissue-specific expression. J Biol Chem.

[B27] Tontonoz P, Nagy L, Alvarez JG, Thomazy VA, Evans RM (1998). PPARgamma promotes monocyte/macrophage differentiation and uptake of oxidized LDL. Cell.

[B28] Rovera G, Santoli D, Damsky C (1979). Human promyelocytic leukemia cells in culture differentiate into macrophage-like cells when treated with a phorbol diester. Proc Natl Acad Sci U S A.

[B29] Naito M, Umeda S, Yamamoto T, Moriyama H, Umezu H, Hasegawa G, Usuda H, Shultz LD, Takahashi K (1996). Development, differentiation, and phenotypic heterogeneity of murine tissue macrophages. J Leukoc Biol.

[B30] Yesner LM, Huh HY, Pearce SF, Silverstein RL (1996). Regulation of monocyte CD36 and thrombospondin-1 expression by soluble mediators. Arterioscler Thromb Vasc Biol.

[B31] Seager Danciger J, Lutz M, Hama S, Cruz D, Castrillo A, Lazaro J, Phillips R, Premack B, Berliner J (2004). Method for large scale isolation, culture and cryopreservation of human monocytes suitable for chemotaxis, cellular adhesion assays, macrophage and dendritic cell differentiation. J Immunol Methods.

[B32] Tangirala RK, Murao K, Quehenberger O (1997). Regulation of expression of the human monocyte chemotactic protein-1 receptor (hCCR2) by cytokines. J Biol Chem.

[B33] Rao A, Luo C, Hogan PG (1997). Transcription factors of the NFAT family: regulation and function. Annu Rev Immunol.

[B34] Macian F, Lopez-Rodriguez C, Rao A (2001). Partners in transcription: NFAT and AP-1. Oncogene.

[B35] Lenny N, Westendorf JJ, Hiebert SW (1997). Transcriptional regulation during myelopoiesis. Mol Biol Rep.

[B36] Clarke S, Gordon S (1998). Myeloid-specific gene expression. J Leukoc Biol.

[B37] Nienhuis AW, Bunn HF, Turner PH, Gopal TV, Nash WG, O'Brien SJ, Sherr CJ (1985). Expression of the human c-fms proto-oncogene in hematopoietic cells and its deletion in the 5q- syndrome. Cell.

[B38] Scheibenbogen C, Andreesen R (1991). Developmental regulation of the cytokine repertoire in human macrophages: IL-1, IL-6, TNF-alpha, and M-CSF. J Leukoc Biol.

[B39] Fiorucci G, Percario ZA, Marcolin C, Coccia EM, Affabris E, Romeo G (1995). Inhibition of protein phosphorylation modulates expression of the Jak family protein tyrosine kinases. J Virol.

[B40] Callus BA, Mathey-Prevot B (1998). Interleukin-3-induced activation of the JAK/STAT pathway is prolonged by proteasome inhibitors. Blood.

